# Supplementary material for: Increased signaling entropy in cancer requires the scale-free property of protein interaction networks
Source: Sci Rep. 2015 Apr 28;5:9646. doi: 10.1038/srep09646 (PMC4412078; doi:10.1038/srep09646)
Supplement: Supplementary Information [file srep09646-s1.pdf]

Supplementary Information for:

Increased signalling entropy in cancer requires the scale-free property of protein interaction networks

Andrew E. Teschendorff, Christopher R. S. Banerji, Simone Severini, Reimer Kuehn and Peter Sollich

Corresponding author: a.teschendorff@ucl.ac.uk

SUPPLEMENTARY FIGURES:

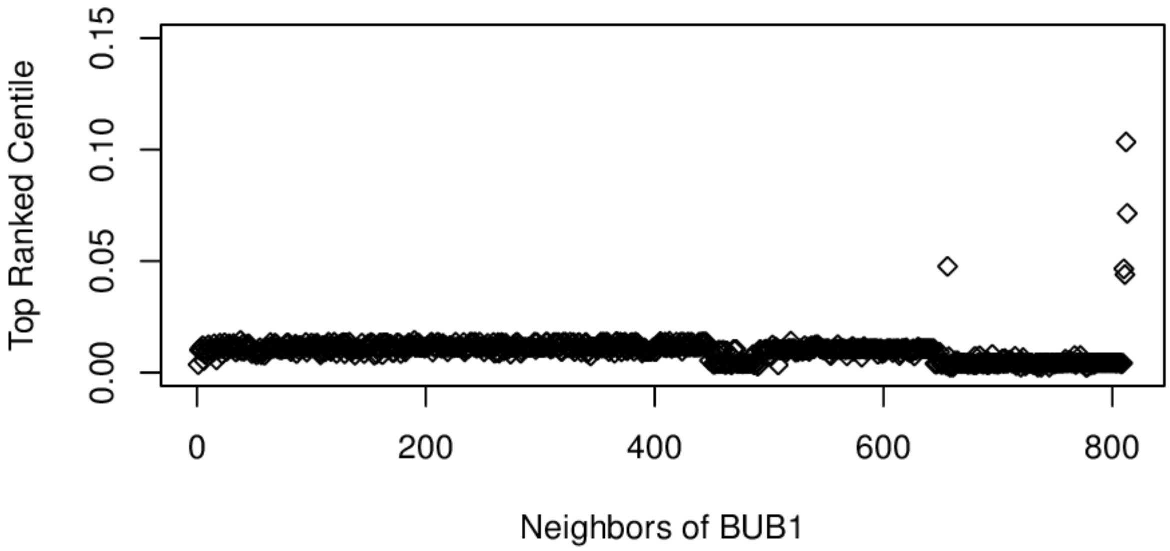

Fig.S1: For each neighbour, *j*, of BUB1 in the PPI network (a total of 813 neighbours, x-axis) we plot the centile (y-axis) at which BUB1 ranks among all neighbours of *j* , where all neighbours of *j* have been ranked according to the fold-change increase in differential expression between normal and liver cancer (TCGA set). We can see that for most of the 813 neighbours, BUB1 consistently ranks among the top 5%.

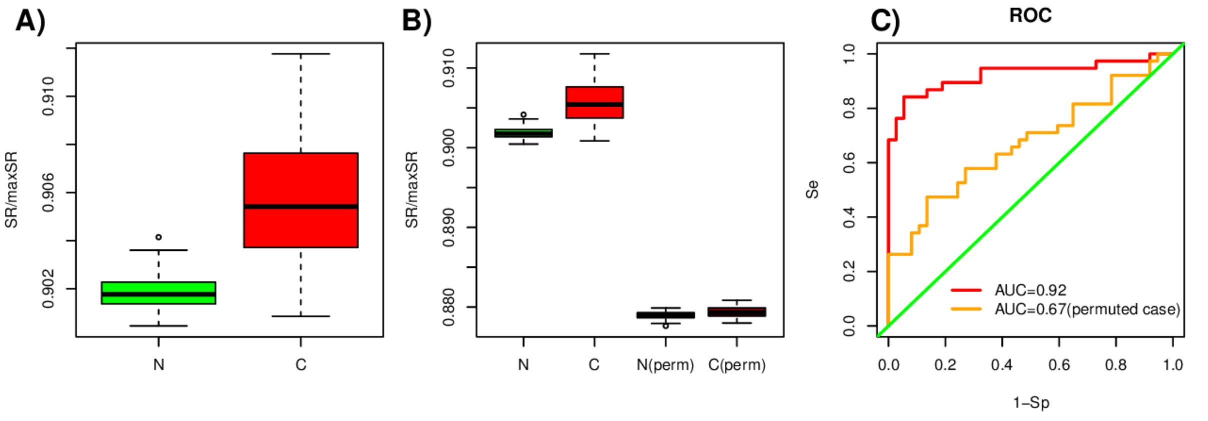

Fig.S2: Figure reproduced from Teschendorff AE et al Methods 2014 (1), demonstrating the **A)** increased entropy rate in liver cancer (n=38) compared to normal liver tissue (n=37) for an Affymetrix gene expression data from Wurmbach et al (2), **B)** how the difference is significantly reduced if expression profiles are permuted over the PPI network, and **C)** the corresponding comparison of the AUC for the unpermuted vs permuted expression profiles.

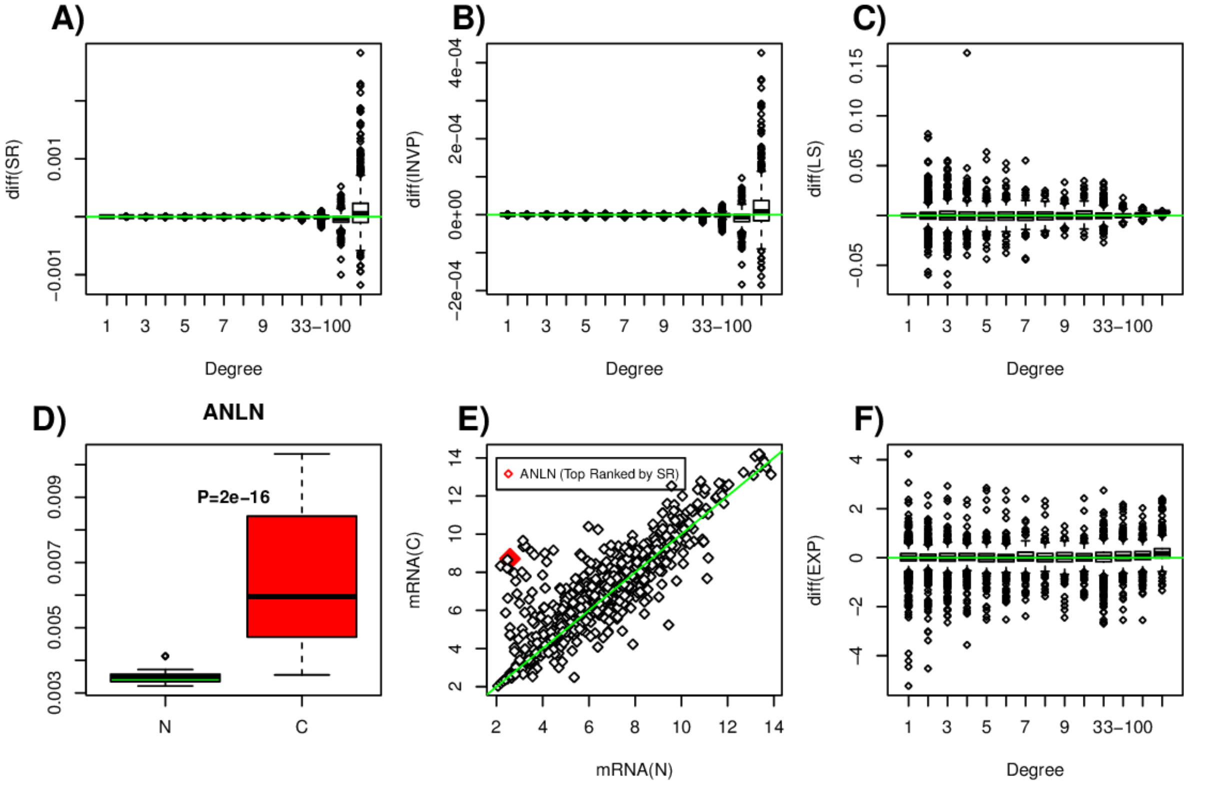

Fig.S3: A) Average difference in the local entropy rate (i.e. the average difference in entropy rate of a given gene, between normal and liver cancer (2)) vs. gene (node) degree in the PPI network. Degrees have been binned into groups of approximately equal size. **B)** As A) but for the invariant measure. **C)** As A) but for the local entropy. **D)** Comparison of the entropy rate contribution of gene ANLN between normal liver and liver cancer (2). ANLN was ranked top in terms of the difference in the entropy rate. **E)** A scatterplot of the expression values of ANLN and its neighbours in a representative cancer sample (y-axis) vs. a representative

normal sample (x-axis). **F)** Plot of the difference in gene expression (log-fold-change) between normal and liver cancer against node-degree. In panels A), B), C) and F) positive values mean higher values in cancer.

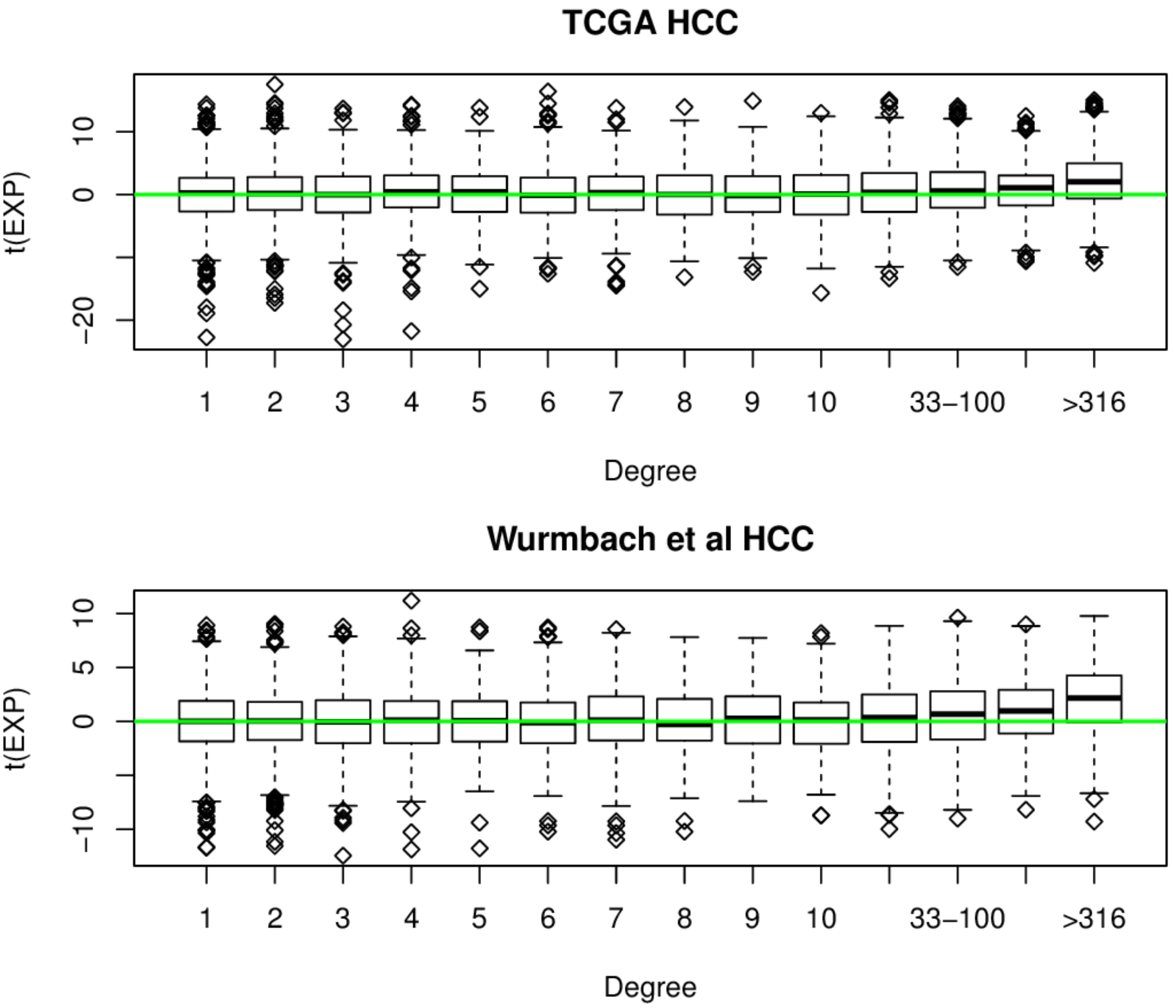

Fig.S4: Plot of the t-statistics of differential expression between normal liver and liver cancer (TCGA and Wurmbach et al (2)) against gene (node degree), with degrees binned into groups of roughly equal size. Note how in both data sets, the highest-degree hubs exhibit preferential increases in gene expression in cancer, whilst genes showing the most significant decreases tend to map to low-degree nodes.

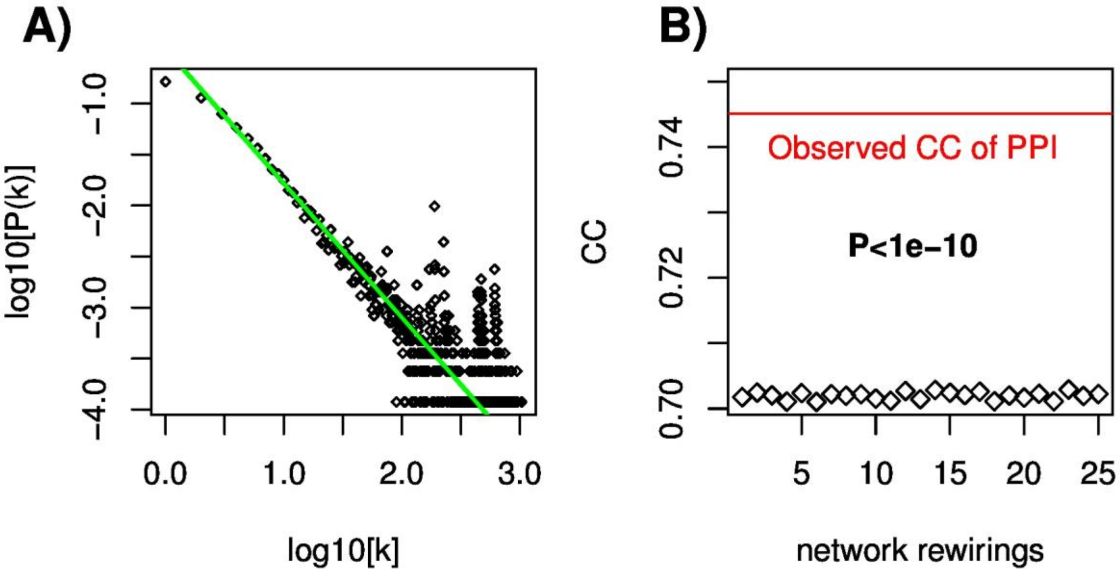

Fig.S5: A) Plot of log10[probability of degree k] against log10[degree] for the full PPI network, demonstrating its approximate scale-freeness. **B)** Comparison of observed clustering coefficient (transitivity) of full PPI network (red line) to the clustering coefficients (CC) of 25 randomly rewired networks of same size and degree distribution as the original PPI.

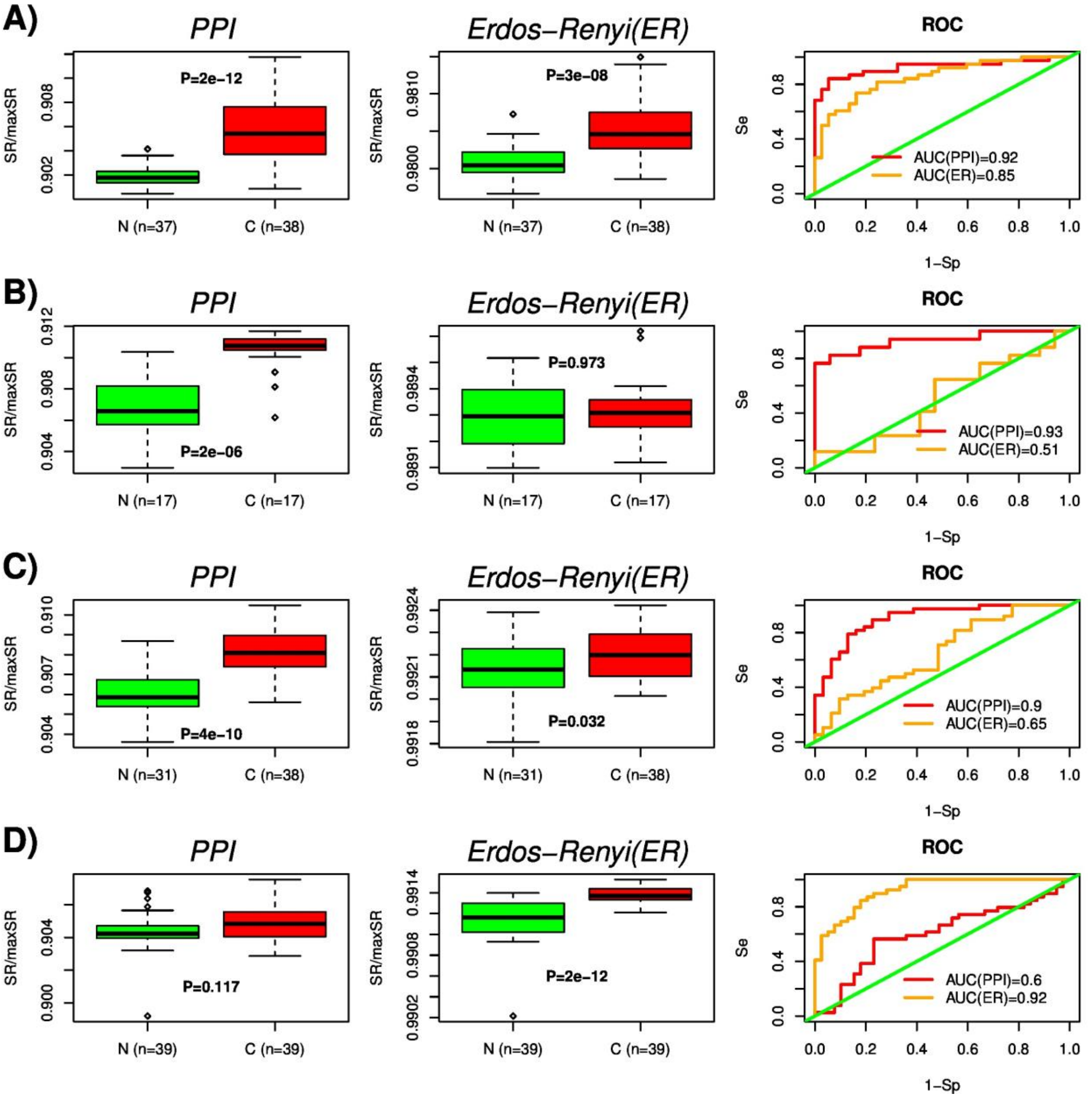

Fig.S6: A) Left panel: Entropy rate difference between normal liver (N) and liver cancer (C) for the Affymetrix gene expression set of Wurmbach et al (2), and for the real PPI network. **Middle panel:** As left panel but now for an equivalent Erdos-Renyi random graph, matched for size and connectivity and preserving the rank correlation between differential expression and node degree as observed in the real PPI network. **Right panel:** Corresponding ROC curves plus AUC values. **B-D)** As A) but for a B) normal/cancer colon, C) normal/cancer gastric and D) normal/cancer pancreatic Affymetrix gene expression data sets, as used previously in (3).

Supplementary References:

1. Teschendorff AE, Sollich P, & Kuehn R (2014) Signalling entropy: A novel network-theoretical framework for systems analysis and interpretation of functional omic data. *Methods* 67(3):282-293.
2. Wurmbach E*, et al.* (2007) Genome-wide molecular profiles of HCV-induced dysplasia and hepatocellular carcinoma. *Hepatology* 45(4):938-947.
3. Banerji CR*, et al.* (2013) Cellular network entropy as the energy potential in Waddington's differentiation landscape. *Scientific reports* 3:3039.
